# A chromosome-scale genome assembly of Timorese crabgrass (*Digitaria radicosa*): a useful genomic resource for the Poaceae

**DOI:** 10.1093/g3journal/jkae242

**Published:** 2024-10-10

**Authors:** Koki Minoji, Toshiyuki Sakai

**Affiliations:** Laboratory of Crop Evolution, Graduate School of Agriculture, Kyoto University, Muko, Kyoto 617-0001, Japan; Laboratory of Crop Evolution, Graduate School of Agriculture, Kyoto University, Muko, Kyoto 617-0001, Japan

**Keywords:** *Digitaria radicosa*, *Digitaria*, crabgrass, genome assembly, synteny, population genetics, NLR

## Abstract

Timorese crabgrass (*Digitaria radicosa*) is a grass species commonly found in Southeast Asia and Oceania. *Digitaria* species have high intraspecific and interspecific genetic and phenotypic diversity, suggesting their potential usefulness as a genetic resource. However, as the only high-quality reference genome available is for a tetraploid *Digitaria* species, a reference genome of the diploid species *D. radicosa* would be a useful resource for genomic studies of *Digitaria* and Poaceae plants. Here, we present a chromosome-level genome assembly of *D. radicosa* and describe its genetic characteristics; we also illustrate its usefulness as a genomic resource for Poaceae. We constructed a 441.6-Mb draft assembly consisting of 61 contigs with an N50 contig length of 41.5 Mb, using PacBio HiFi long reads. We predicted 26,577 protein-coding genes, reaching a Benchmarking Universal Single-Copy Orthologs score of 96.5%. To demonstrate the usefulness of the *D. radicosa* reference genome, we investigated the evolution of *Digitaria* species and the genetic diversity of Japanese *Digitaria* plants based on our new reference genome. We also defined the syntenic blocks between *D. radicosa* and 2 Poaceae crops, fonio and rice, and the diverse distribution of representative resistance genes in *D. radicosa*. The *D. radicosa* reference genome presented here should help elucidate the genetic relatedness of *Digitaria* species and the genetic diversity of *Digitaria* plants. In addition, the *D. radicosa* genome will be an important genomic resource for Poaceae genomics and crop breeding.

## Introduction

Timorese crabgrass (*Digitaria radicosa*) is a diploid (2*n* = 18) plant commonly found in Southeast Asia and Oceania. The genus *Digitaria* comprises over 250 species in the family Poaceae, distributed mostly in tropical and warm temperate regions around the world (POWO 2024). *Digitaria* species are mainly considered to be agricultural weeds, with 17 species registered as invasive plant species in CABI (2024). Nonetheless, several species are useful resources for agriculture. For example, fonio (*Digitaria exilis*) is an African indigenous cereal crop with highly nutritious grains, drought tolerance, and adaptation to nutrient-poor soils (Ayenan *et al.* 2018). Iburu (*Digitaria iburua*) and raishan (*Digitaria compacta*) are 2 other indigenous grain crops (Jideani 1999; Prance and Nesbitt 2004). Pangola grass (*Digitaria eriantha*) and hairy crabgrass (*Digitaria sanguinalis*) are utilized for livestock pastures and to control soil erosion (Pitman *et al.* 2016; Weinert-Nelson *et al.* 2022). Thus, *Digitaria* species can be weeds, food, or pasture crops, underscoring the interspecific phenotypic diversity of this genus. Therefore, understanding the genetic relatedness of *Digitaria* species may help to elucidate the weediness of Poaceae species and improve the utilization of *Digitaria* species in agriculture.

*Digitaria* plants also show high intraspecific genotypic and phenotypic diversity (Tsuyuzaki 2005; Scarcelli *et al.* 2011; Abrouk *et al*. 2020; Fukano *et al*. 2020; Wang *et al*. 2021). An exploration of the relationship between phenotypes and genotypes of diversified *Digitaria* populations will help determine the genetic architecture of various Poaceae traits. However, only a single chromosome-scale reference genome for a *Digitaria* species is currently available, for the cultivated tetraploid *Digitaria* species *D. exilis* (Abrouk *et al.* 2020; Wang *et al.* 2021). The polyploidy of this reference genome and the limited number of reference genomes hamper efforts to perform genomic analyses of *Digitaria* species (Ravet *et al.* 2018; Gonçalves Netto *et al.* 2021). In addition, *Digitaria* species have previously been classified using morphology-based approaches, and the phylogenetic relationship has been estimated from few DNA markers (Vega *et al.* 2009; Soreng *et al.* 2022; Touafchia *et al.* 2023). Notably, these 2 systems do not agree; a consensus phylogenetic relationship remains unclear. Therefore, a reference genome for a diploid *Digitaria* species will facilitate genomic analysis, including population genomics and evolutionary analysis, to understand the genetic relatedness of *Digitaria* species and the genetic contribution to phenotypic diversity (Ravet *et al.* 2018).

*Digitaria* plants commonly grow alongside rice at the edge of paddy fields in Japan (Supplementary Fig. 1). Rice (*Oryza sativa*) and *Digitaria* are both devastated by blast disease caused by the fungus *Magnaporthe* spp (Ou 1980). Notably, there is host plant specificity, with blast fungi isolated from *Digitaria* being *Magnaporthe grisea*, while isolates from rice are *Magnaporthe oryzae* (Tosa and Chuma 2014). Host specificity is determined by the interaction between effector proteins secreted by the fungus and resistance genes from the host plant (Tosa and Chuma 2014; Yoshida *et al.* 2016). Effectors secreted by *M. oryzae* are recognized by nucleotide-binding domain and leucine-rich repeat (NLR) proteins, which activate the resistance response (Cesari *et al.* 2013; Maqbool *et al.* 2015). Previous studies have shown how domains within NLRs, named integrated domains (IDs), are involved in effector recognition specificities (Kroj *et al.* 2016; Marchal *et al.* 2022). *Digitaria* species inhabiting the same niche as rice plants may, therefore, have NLRs with IDs that recognize effectors secreted by *M. oryzae* isolates. Genomic resources for Japanese *Digitaria* species may, thus, provide resistance genes that can be deployed in rice against *M. oryzae*.

In this study, we constructed a highly contiguous and high-quality reference genome for the diploid species *D. radicosa*. We performed an evolutionary analysis using sequencing data from multiple *Digitaria* species and a population genetic analysis using *D. radicosa* and *Digitaria ciliaris* collected in Japan, based on our new reference genome. We also identified syntenic blocks between *D. radicosa* and Poaceae crops and the distribution of NLR genes in *D. radicosa*. These results highlight the utility of this new chromosome-scale diploid *Digitaria* reference genome for Poaceae genomics.

## Methods and materials

### Sample collection

To generate the reference genome, 1 *D. radicosa* plant was collected from Mozume, Kyoto Prefecture, Japan, in August 2022 and kept in a growth chamber at 32°C with a 12/12-h light/dark cycle. Ten *D. radicosa* plants were sampled for population genomics from Kansai region, Japan: 4 from Kyoto, 2 from Kobe, 1 from Wakayama, and 3 from Shirahama (Supplementary Table 1), in September 2023. Eleven *D. ciliaris* plants were also sampled for population genomics from paddy fields, coastal areas, and park areas in September 2023: 5 from Kyoto and 6 from Wakayama, Japan (Supplementary Table 1; Supplementary Fig. 1). The collected samples were identified following dichotomous keys given by Ohashi *et al*. (2016). These keys are also given by Chen and Phillips (2006), Verloove (2008), and Boonsuk *et al*. (2016). We distinguished *D. radicosa* and *D. ciliaris* based on the habitat, color of the lemma, the number of racemes, and the morphology of raceme and spikelet (Supplementary Fig. 2).

### DNA isolation, library construction, and genome sequencing

For sequencing by HiFi reads, genomic DNA was extracted from 1 *D. radicosa* plant using 1 g of lyophilized leaf tissue and Qiagen Genomic-tips 20/G. The DNA was then size selected using a Short Read Eliminator XS Kit (PacBio) and g-TUBEs (Covaris). HiFi libraries were constructed using a SMRTbell Express Template Prep Kit 2.0 (PacBio); the resulting HiFi libraries were sequenced on a PacBio Sequel II instrument. For short-read sequencing, genomic DNA was extracted from the same *D. radicosa* plant using 100 mg of lyophilized leaf tissue and a Maxwell RSC Genomic DNA Kit (Promega). Sequencing libraries were constructed using a MGIEasy PCR-Free DNA Library Prep Set (MGI); the resulting libraries were sequenced on a DNBSEQ-G400 instrument as paired-end 150-bp reads. For population genomic analysis, a MIG-seq library was constructed. Genomic DNA was extracted from *D. radicosa* and *D. ciliaris* plants using 20 mg of young leaf tissue with a DNeasy Plant Mini Kit (Qiagen). After the DNA concentrations were adjusted to 10 ng/μL, the first PCR was performed using a Multiplex PCR Assay Kit Ver.2 (Takara) with MIG-seq primer set-1 developed by Suyama and Matsuki (2015). The PCR program followed Nishimura *et al.* (2022) with the exception of the initial denaturation, performed at 94°C for 1 min. The first PCR products were diluted 50-fold in ultrapure water. Indexing primers were added to each sample during the second PCR, which was performed with PrimeSTAR GXL DNA Polymerase (Takara) following the PCR program of Nishimura *et al*. (2022) but with a final extension of 7 min at 68°C. The concentration of each amplicon was measured and adjusted before pooling equal amounts of DNA from each sample. The pooled library was purified using AMPure XP Beads (Beckman Coulter). The library was then size selected using SPRIselect (Beckman Coulter). The bead ratios for the left size selection and right size selection were set to 0.75× and 0.56×, respectively. The library was sequenced on an Illumina NovaSeq platform as paired-end 150-bp reads by Nippon Genetics Co., Ltd.

### Genome assembly and evaluation

Genome size was estimated within the MaSuRCA pipeline based on the k-mer abundance distribution using the short reads generated on the DNBSEQ instrument (Zimin *et al.* 2013). Primary contigs were assembled using PacBio HiFi reads by the hifiasm v0.19.8 assembler (Cheng *et al.* 2021). The primary genome assembly was polished using HiFi reads and short reads by NextPolish2 v0.2.0 (Hu *et al.* 2024). To remove redundant sequences, purge_dups v1.2.5 was used (Guan *et al.* 2020). The assembled contigs were then scaffolded into pseudochromosomes by mapping against the chromosome-scale *D. exilis* genome, using Ragtag v2.1.0 (Abrouk *et al.* 2020; Alonge *et al.* 2022). To assess the completeness of the assembled genome, a Benchmarking Universal Single-Copy Orthologs (BUSCO) v5.6.1 analysis was performed with the poales_odb10 dataset (Manni *et al.* 2021).

### Genome annotation

The *D. radicosa* genome was annotated using the RepeatModeler2 pipeline, which identifies repeats in the genome, and the BRAKER3 pipeline for de novo gene prediction, homology-based predictions, and transcriptome-based predictions to predict protein-coding genes (Flynn *et al.* 2020; Gabriel *et al.* 2024). For the annotation of repeat elements, RepeatModeler2 v2.0.5 was used to perform ab initio prediction with default parameters (Flynn *et al.* 2020). The annotated repeats were then filtered to only keep complex repeats for soft masking. Repeat elements across the genome were masked before gene annotation by RepeatMasker v4.1.5 (Tarailo-Graovac and Chen 2009). Next, BRAKER3 was used to train GeneMark-ETP and AUGUSTUS on the soft-masked genome. For transcriptome-based gene annotation, 14 public transcriptome deep sequencing (RNA-seq) libraries generated for *Digitaria* species were downloaded from National Center for Biotechnology Information (NCBI) and mapped to the *D. radicosa* genome using STAR v2.7.10a to generate mapped BAM files (Supplementary Table 2) (Dobin *et al.* 2013). For homology-based gene annotation, protein sequences from *Arabidopsis thaliana* (GCA_000001735.2), *Setaria italica* (GCA_000263155.2), *D. exilis* (GCA_015342445.1), *Sorghum bicolor* (GCA_000003195.3), *O. sativa* (GCA_001433935.1), and *Zea mays* (GCF_902167145.1) were downloaded from NCBI. To validate the quality of predicted gene sets, completeness was assessed using BUSCO v5.6.1 with the poales_odb10 dataset (Manni *et al.* 2021). Completeness and lineage consistency of the proteome (the proportion of protein sequences placed into known gene families from the same lineage) were also assessed using OMArk (Nevers *et al.* 2024).

### Phylogenetic analysis

Publicly available sequencing reads for the genomes and transcriptomes of *Digitaria* species (*Digitaria abyssinica*, *Digitaria bicornis*, *Digitaria biformis*, *Digitaria brownii*, *Digitaria californica*, *D. ciliaris*, *Digitaria ctenantha*, *Digitaria cuyabensis*, *Digitaria didactyla*, *D. eriantha*, *D. exilis*, *Digitaria fuscescens*, *Digitaria longiflora*, *Digitaria milanjiana*, *Digitaria monodactyla*, *Digitaria pseudodiagonalis*, *D. sanguinalis*, and *Digitaria ternata*) were used for phylogenetic analysis. The accession numbers for all raw reads are listed in Supplementary Table 2. The obtained DNA-seq and RNA-seq reads were filtered and trimmed using fastp (Chen *et al.* 2018). The quality-trimmed DNA-seq reads were mapped to the new *D. radicosa* reference genome assembled in this study using bwa-mem2 (Vasimuddin *et al.* 2019). RNA-seq reads were mapped using STAR v2.7.10a (Dobin *et al.* 2013). Variant calling was performed using GATK according to GATK Best Practices recommendations (McKenna *et al.* 2010). Single-nucleotide polymorphisms (SNPs) at biallelic sites with read depth > 1 in all samples were used for phylogenetic analysis (Supplementary File 1). A maximum-likelihood phylogenetic tree was estimated using RAxML (Stamatakis 2006). The GTRCAT model was selected as the best substitution model based on the Akaike information criterion calculated by ModelTest-NG (Darriba *et al.* 2020).

### Population genetics

The reads obtained from the MIG-seq library were used for population genetic analysis. The raw reads were trimmed and quality filtered using fastp v0.23.2 (Chen *et al.* 2018). The trimmed reads were mapped to the new *D. radicosa* genome assembled in this study using BWA v0.7.17 (Li 2013). Alignments with mapping quality below 40 and reads with multiple alignments were removed using SAMtools v1.19.2 (Li *et al*. 2009). Each bam file containing reads from a single sample was combined to a single bam file, and variant calling was performed using bcftools v1.16 (Li 2011). Three variant call format (VCF) files were created, containing SNPs from *D. radicosa*, *D. ciliaris*, or both species (Supplementary File 2). Filtering of SNPs was performed on the number of alleles (only 2-allele loci were retained), minor allele frequency (≥0.05), genotyping quality (≥20), and read depth (within a range of 5–50) using bcftools and VCFtools v0.1.17 (Danecek *et al.* 2011). SNPs within possible linkage disequilibrium regions were then pruned based on (1) physical distance between SNPs using the bcftools “+prune” plugin with options “-n 1 -w 1kb” and (2) genotypic correlation between SNPs using the PLINK version 1.90 “—indep-pairwise” option with settings “5000 kb 1 0.1” (Chang *et al.* 2015). Using these filtered SNP datasets, a principal component analysis (PCA) was performed using the PLINK “—pca” option. The result of PCA with all samples suggested the misidentification of 2 samples as *D. ciliaris*, so these 2 samples were removed before repeating the analysis (Supplementary Fig. 3).

### Synteny analysis

Reference-guided assembly possibly introduces errors as structural variations. Therefore, we used the contigs of the first assembly for synteny analysis. MCScan was used to define syntenic blocks between the *D. exilis* and *D. radicosa* genomes and between the *O. sativa* and *D. radicosa* genomes (Wang *et al.* 2012; Sakai *et al.* 2013; Abrouk *et al.* 2020). We used a rice reference genome of Nipponbare cultivar (IRGSP-1.0) (Sakai *et al.* 2013). MCScan was performed and visualized using JCVI python library with default parameters (Tang *et al*. 2024).

### NLR annotation and phylogenetic analysis

NLRtracker was used to identify NLR genes from the new *D. radicosa* genome (Kourelis *et al.* 2021). These NLRs from *D. radicosa* and functionally validated NLRs from Poaceae species in the RefPlantNLR database were used for phylogenetic analysis (Kourelis *et al.* 2021). NLR protein sequences were aligned using MAFFT v.7, deleting the gaps in the alignments (Katoh and Standley 2013). Sequences of NLRs with truncated NB-ARC domains were manually removed. The sequences of the NB-ARC domains from the aligned NLR proteins were used to reconstruct phylogenetic trees (Supplementary File 3). A maximum-likelihood phylogenetic tree was generated in RAxML version 8.2.12 using the JTT model. The bootstrap values in the resulting tree were based on 100 iterations (Stamatakis 2006).

## Results

### Genome assembly and genome annotation

We assembled a reference genome for Timorese crabgrass (*D. radicosa*, 2*n* = 18) using PacBio HiFi long reads and DNBSEQ short reads. To estimate the size of the *D. radicosa* genome, we analyzed 88.3 Gb of short reads based on the k-mer abundance distribution, revealing an estimated genome size of 420 Mb. To obtain the reference genome, we assembled 21.7 Gb of HiFi long reads and polished the resulting contigs with 88.3 Gb of DNBSEQ short reads. We obtained 61 contigs covering 441.6 Mb in total length with a contig N50 value of 41.5 Mb. The length of the longest contig was 66.2 Mb, indicating that some contigs were assembled at the scale of a chromosome. These assembly statistics reflect the good contiguity and integrity of our genome assembly (Table 1). To estimate the chromosomes that contigs derive from, we performed reference-guided assembly using the *D. exilis* genome. As a result, 30 contigs encompassing 440.8 Mb of sequence were anchored to 9 pseudochromosomes (Supplementary Table 3). The GC content of the genome was 45.4% (Table 1). To evaluate the quality of the assembled genome, we performed a BUSCO analysis using the Poales lineage gene dataset. We calculated a BUSCO score of 96.3% (4,714 out of 4,896) for the newly assembled genome, with 93.4% single-copy and 2.9% duplicated genes (Table 1). These results indicate that the chromosome-scale assembled *D. radicosa* genome in this study is highly contiguous and of high quality.

**Table 1.** Summary statistics for genome assembly and genome annotation.

Item	Category	Number
Sequencing	PacBio HiFi reads	21.7 Gb
	DNBSEQ short reads	88.3 Gb
Assembly	Estimated genome size	420.3 Mb
	Assembled genome size	441.6 Mb
	Contig number	61
	Contig N50	41.5 Mb
	Longest contig	66.2 Mb
	Complete BUSCOs (poales_odb10)	96.3% (4,714/4,896)
		S: 93.4%, D: 2.9%, F: 0.3%, M: 3.4%
Annotation	GC content	45.4%
	Repeat sequences	50.1%
	Number of protein-coding genes	26,577
	BUSCO score (poales_odb10)	95.3% (4,665/4,896)
		S: 92.6%, D: 2.7%, F: 0.2%, M: 4.5%

D, duplicated; F, fragmented; M, missing; S, single copy.

To predict protein-coding genes in the genome, we performed an ab initio prediction of repeat elements to mask them in the genome, after which we subjected the masked genome to our gene prediction pipeline. We identified 221.4 Mb of repetitive elements in the *D. radicosa* genome, representing 50.1% of the entire genome (0.1% of short interspersed nuclear elements, 1.6% of long interspersed nuclear elements, 16.5% of long terminal repeat elements, 3.7% of DNA transposons, and 28.3% of unclassified repeats) (Supplementary Table 4). The gene prediction pipeline identified 26,577 protein-coding genes (Table 1). We assessed the completeness of the gene prediction by repeating the BUSCO analysis with the same Poales lineage gene dataset. We identified 95.3% (4,665 out of 4,896) complete BUSCO genes among the predicted protein-coding genes, with 92.6% as single copies and 2.7% as duplicated genes (Table 1). We also performed an analysis with the OMArk software, which returned a level of completeness of 90.6% (18,575 out of 20,501) and a taxonomic consistency of 93.2% (24,775 out of 26,577) (Supplementary Table 5). These results indicate the high completeness and accuracy of our predicted gene set.

### Evolutionary analysis of *Digitaria* species

**Fig. 1.**
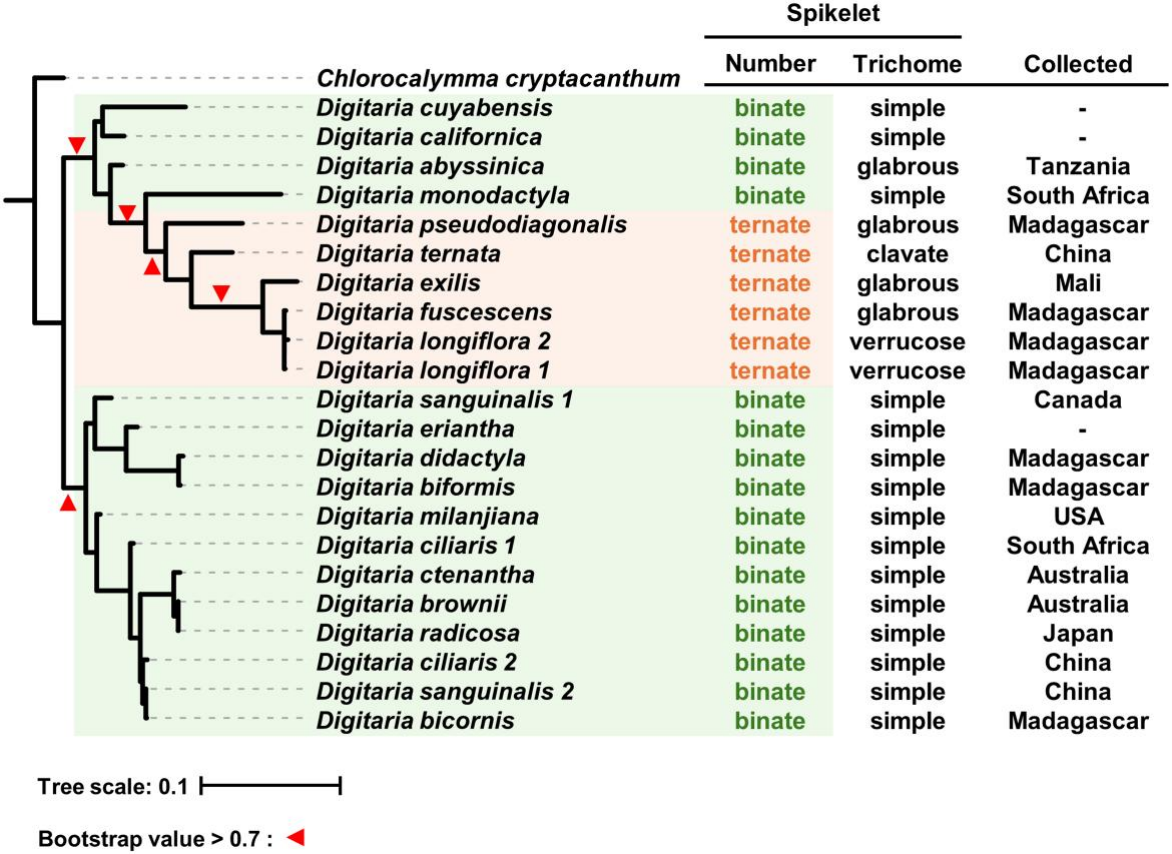
The phylogenetic relationships of *Digitaria* species reflect their spikelet morphology. Phylogenetic tree of 19 *Digitaria* species and 1 *Chlorocalymma* species as an outgroup. The maximum-likelihood phylogenetic tree was generated in RAxML version 8.2.12 with the JTT model using 2,052 SNPs. The scale bar indicates the evolutionary distance in nucleotide substitution per site. The well-supported nodes are visualized as arrowheads with bootstrap values > 0.7 based on 1,000 iterations. The right table indicates the number of spikelets at each node on the rachis, the trichome morphology, and country where each sample was collected.

The morphological classification and current estimated phylogenetic relationship of *Digitaria* species are not consistent (Clayton *et al.* 2006; Touafchia *et al.* 2023; POWO 2024). However, the issues of relatedness among *Digitaria* species should be solved by a genomic analysis based on high-quality genome data. To investigate the genetic relatedness of *Digitaria* species, we thus performed a phylogenetic analysis based on our new *D. radicosa* reference genome using publicly available DNA-seq and RNA-seq data for 22 samples from 19 *Digitaria* species (Supplementary Table 2). We used *Chlorocalymma cryptacanthum* as an outgroup. We aligned the publicly available DNA-seq and RNA-seq reads to our reference genome and identified 2,052 SNPs with a read depth over 1 for all 23 samples. The resulting phylogenetic tree reconstructed from the obtained SNPs showed that *Digitaria* species can be classified into 2 well-supported clades (Fig. 1). The first clade contains a cultivated *Digitaria* species, *D. exilis*, and its putative wild progenitor, Indian crabgrass (*D. longiflora*). The second clade contains Asian weedy *Digitaria* species including *D. ciliaris* and *D. radicosa*. While the close relationship of *D. exilis* and *D. longiflora* was known (Abrouk *et al.* 2020), we noticed that *D. fuscescens* is also phylogenetically closely related to *D. exilis* in our phylogenetic analysis (Fig. 1).

Our phylogenetic tree included multiple samples for 3 *Digitaria* species. The 2 *D. longiflora* plants collected from Madagascar had almost identical genotypes (Fig. 1). However, the 2 *D. sanguinalis* plants collected from Canada or China and the *D. ciliaris* plants collected from China or South Africa were phylogenetically more diverse but still fell within the same clade (Fig. 1).

To compare the genetic relationship and the phenotypic classification, we investigated spikelet morphology, which is traditionally used for the grouping of *Digitaria* species (Henrard 1950). The number of spikelets at each node on the rachis was clearly different among the 2 clades defined by the phylogenetic tree (Fig. 1). Five *Digitaria* species (*D. pseudodiagonalis*, *D. ternata*, *D. exilis*, *D. fuscescens*, and *D. longiflora*) constituted a well-supported clade with ternate spikelets (Fig. 1). Other species showed binate spikelets (Fig. 1). Looking at trichome morphology of spikelets, *Digitaria* species from the second clade (*D. sanguinalis* to *D. bicornis*) had simple hair form. By contrast, *Digitaria* species from the first clade (*D. cuyabensis* to *D. longiflora*) showed wide variation in their spikelet trichome morphology (Fig. 1) (Boonsuk *et al*. 2016).

### Genetic diversity of *Digitaria* species in Japan

**Fig. 2.**
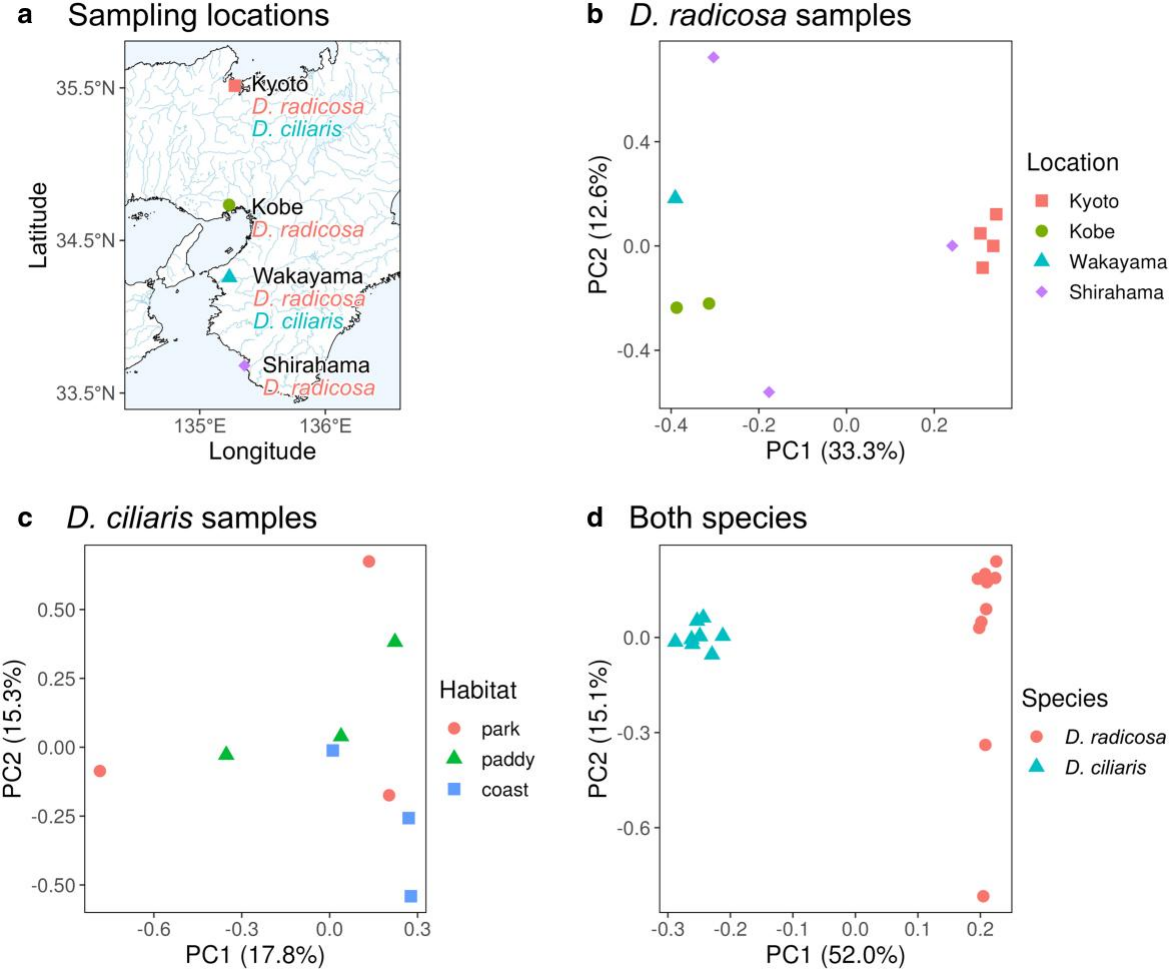
Population genetic analysis of *D. radicosa* and *D. ciliaris*. a) Sampling locations of *D. radicosa* and *D. ciliaris* plants. The map is based on Global Map Japan (2016). b–d) PCA for *D. radicosa* plants sampled from 4 locations b), *D. ciliaris* plants sampled separately from different habitats at each sampling location c), and plants from both species d). The numbers in parentheses indicate the percentage of standing variance explained by each PC.

Considering the wide geographical distribution of *Digitaria* species in Japan and the reported phenotypic variation among *D. ciliaris* plants, we hypothesized that this species may comprise genetically distinct subpopulations (Kataoka *et al.* 1986; Tsuyuzaki 2005; Fukano *et al.* 2020). To investigate the genetic differentiation among geographically distinct areas and habitats, we performed a PCA using SNPs obtained from 10 *D. radicosa* plants and 11 *D. ciliaris* plants collected from the Kansai region, Japan (Fig. 2a). We obtained *D. radicosa* samples from all 4 locations to reveal any underlying population structure associated with geographical distribution. We also collected *D. ciliaris* samples from 3 habitats (parks, paddy fields, and coastal areas) to investigate genetic variation among plants growing in different habitats (Supplementary Fig. 1). The PCA showed that 2 plants were likely misidentified as *D. ciliaris*, which we removed for the downstream analysis (Supplementary Fig. 3). A total of 206 and 245 SNPs were identified from among plant samples belonging to *D. radicosa* (10 samples) and *D. ciliaris* (9 samples), respectively, and 101 SNPs were identified from among all samples from both species.

The *D. radicosa* plants formed 2 clusters along principal component 1 (PC1), which explained 33.3% of the standing genomic variation (Fig. 2b). Plants collected from Kyoto and Kobe formed distinct clusters according to their respective locations. Plants collected from Shirahama were not clustered based on PCs. These results indicate little genetic variation among plants from Kyoto or Kobe and large genetic variation among plants from Shirahama.

*D. ciliaris* plants collected from different habitats showed a loose clustering pattern (Fig. 2c). Plants from different habitats partially overlapped in the PCA plot, although plants collected from paddy fields and coastal areas appeared to be separated along PC2. Plants collected from parks showed the largest variation along PC1 and PC2.

A PCA plot using all *D. radicosa* and *D. ciliaris* plants supported the clear genomic separation of these 2 species (Fig. 2d). Indeed, the 2 species formed distinct clusters along PC1, explaining 52.0% of the total genomic variation. While all *D. ciliaris* plants were contained within a single cluster, 2 *D. radicosa* plants were distant from the other 8 plants of the same species along PC2 (Fig. 2d).

### Synteny between *D. radicosa* and 2 Poaceae crops

**Fig. 3.**
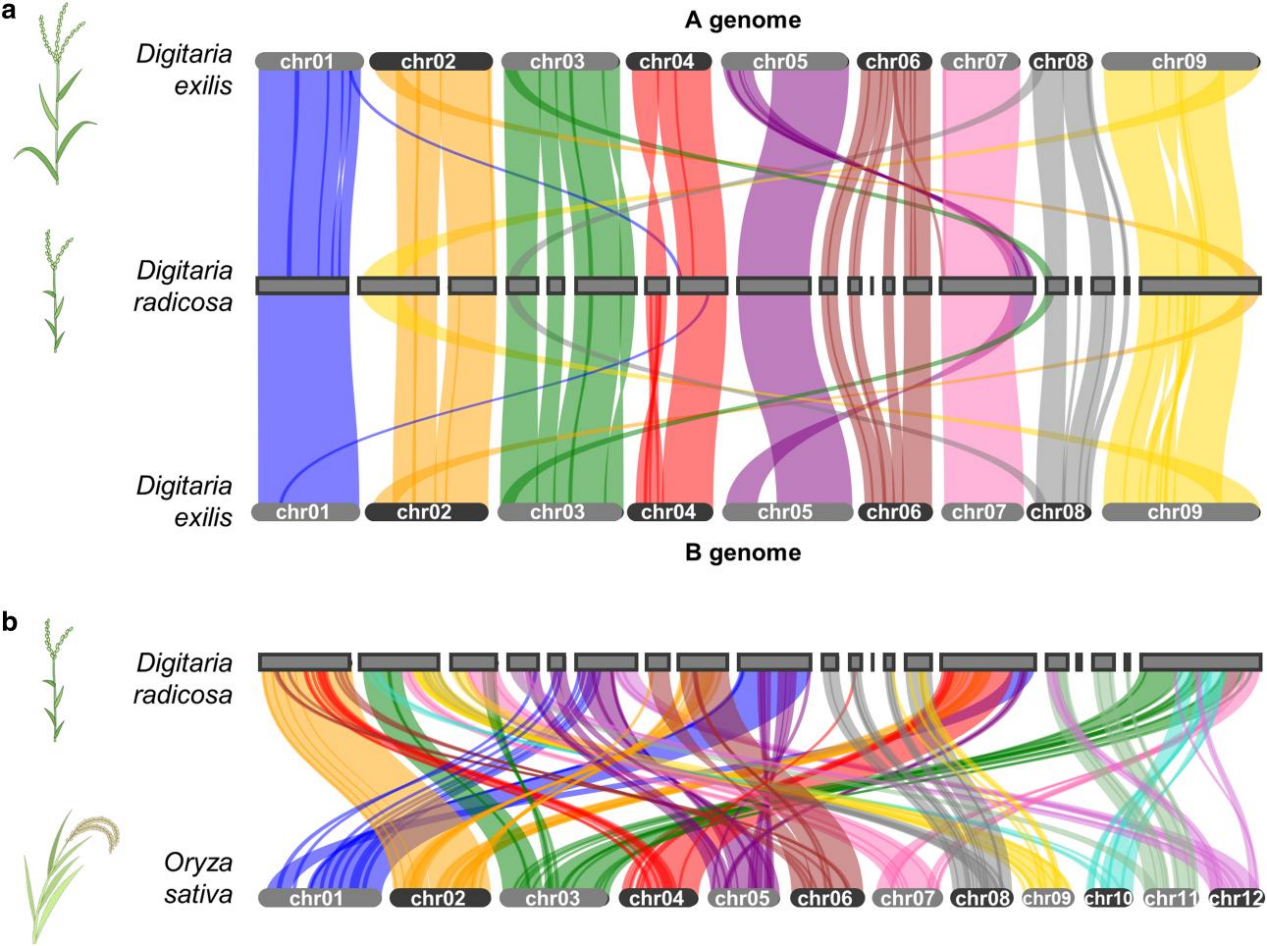
The *D. radicosa* reference genome is highly syntenic with the *D. exilis* and rice genomes. a) The top plot shows the syntenic blocks between *D. radicosa* and the A or B genome of *D. exilis*. b) The bottom plot shows the syntenic blocks between *D. radicosa* and Nipponbare cultivar of rice. Syntenic blocks were identified by MCScan.

Since the only available chromosome-scale genome assembly is for the cultivated *Digitaria* species *D. exilis*, genomic structural variation between wild and cultivated *Digitaria* species was not investigated (Abrouk *et al.* 2020; Wang *et al.* 2021). To explore the presence of structural variation and the degree of synteny between wild *Digitaria* and cultivated *Digitaria* species, we performed a synteny analysis between *D. radicosa* and *D. exilis* using their respective high-quality, chromosome-scale genome assemblies. We also identified syntenic blocks between *D. radicosa* and rice to investigate conserved genomic regions between the 2 Poaceae species. Synteny analysis revealed a high degree of synteny between contigs of *D. radicosa* and all chromosomes of *D. exilis*. Six contigs in *D. radicosa* showed syntenic blocks with over 2 chromosomes of *D. exilis*, suggesting translocations between *D. radicosa* and *D. exilis* (Fig. 3a). A similar analysis between the *D. radicosa* and rice genomes revealed several contig-scale conserved syntenic blocks, exemplified by the syntenic block between a *D. radicosa* contig and chromosome 2 of rice (Fig. 3b). Other *D. radicosa* contigs also showed sequence conservation with some genomic regions in the rice genome, although these conserved sequences were spread across several chromosomes (Fig. 3b).

### Resistance genes in the *D. radicosa* genome

**Fig. 4.**
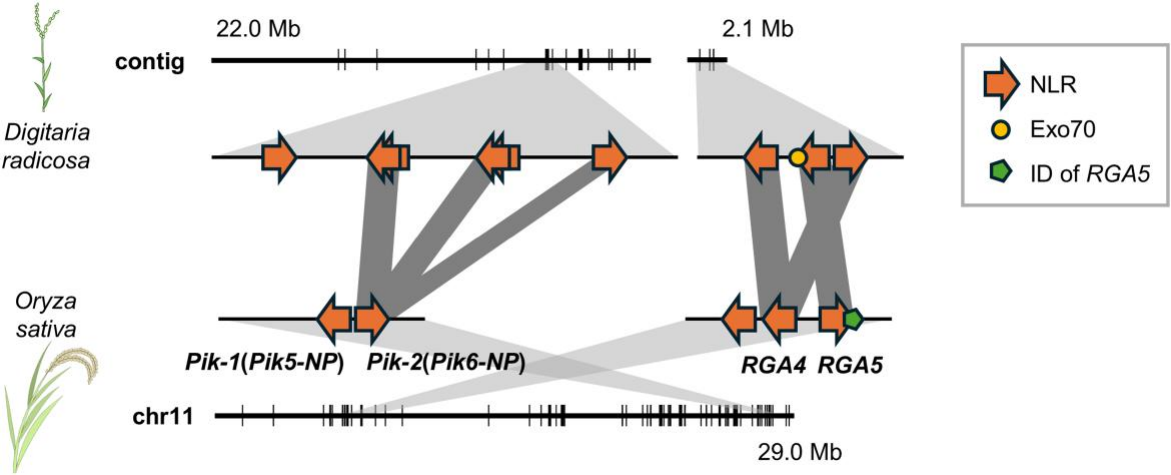
Examples of functionally validated rice NLR orthologs in *D. radicosa.* Top: contigs of *D. radicosa*. Bottom: chromosome 11 of rice. Annotated NLRs are displayed as vertical lines. Middle: genomic regions of *D. radicosa* carrying NLR genes orthologous to *Pik-2* (*Pik6-NP* in Nipponbare) and *RGA4/5* from rice. Arrows indicate NLRs; the circle and pentagon indicate IDs, Exo70 and various IDs in rice *RGA5*. The bands between NLRs indicates phylogenetically well-supported orthologs.

NLR proteins recognize effectors secreted by blast fungus and induce an immune response in Poaceae species including rice (Jones *et al.* 2016). Previous studies showed extra domains of NLRs, named IDs, are involved in effector recognition specificities (Kroj *et al.* 2016; Marchal *et al.* 2022). We hypothesized that *Digitaria* species might have NLRs with IDs recognizing blast fungus isolates from rice plants. To investigate NLRs in *D. radicosa*, we extracted 155 genes annotated as NLR genes from our reference genome and performed a phylogenetic analysis on their NB-ARC domain sequences to classify them, together with 100 functionally validated NLR proteins from 14 other Poaceae species. We determined that *D. radicosa* NLRs are phylogenetically diverse (Supplementary Fig. 4). Combined with the results of synteny analysis, we identified *D. radicosa* genes orthologous to the rice NLR genes *RESISTANCE GENE ANALOG 5* (*RGA5*) and *RGA4*, as well as orthologs for *PYRICULARIA ORYZAE RESISTANCE K-2* (*Pik-2*) on the contigs of *D. radicosa* (Fig. 4) (Kanzaki *et al.* 2012; Cesari *et al.* 2013). These rice NLRs recognize effectors secreted by *M. oryzae* and induce immune responses in rice. In addition, *RGA5* contains a wide variety of external domains in its C terminus as an ID (Shimizu *et al*. 2022). Interestingly, 2 putative *RGA5* ortholog in *D. radicosa* harbored an Exo70 domain that was not observed in rice *RGA5* (Fig. 4).

## Discussion

We generated a chromosome-scale reference genome assembly of the diploid species *D. radicosa*. We obtained 61 contigs with an N50 contig length of 41.5 Mb and BUSCO scores supporting the high contiguity and integrity of this new genome assembly (Table 1). The only previously available chromosome-scale reference genome was from a tetraploid *Digitaria* species, *D. exilis*, with no reference genome for a diploid *Digitaria* species (Abrouk *et al.* 2020; Wang *et al.* 2021). Most standard tools for genomic analysis have been developed for diploid genomes and are not easily applied to polyploid genomes (Dufresne *et al.* 2014). Therefore, our assembled diploid *Digitaria* genome will allow population genetic analysis and evolutionary analysis of *Digitaria* species.

To verify the utility of our newly assembled diploid *Digitaria* genome, we estimated the genetic relatedness of 19 *Digitaria* species (Fig. 1). For instance, the species *D. monodactyla* with binate spikelets was previously classified together with species having ternate spikelets (Touafchia *et al.* 2023). However, our phylogenetic tree based on genome-wide SNPs demonstrated that *Digitaria* species with ternate spikelet morphology constitute 1 well-supported clade, suggesting a clear genetic difference between species with ternate and binate spikelet morphology (Fig. 1; Supplementary Fig. 5). Therefore, our evolutionary analysis based on diploid *Digitaria* species possibly provides a more precise phylogeny of *Digitaria* species. In addition, our phylogenetic tree showed distant genetic relatedness among some plants from the same species collected from different geographical locations (Fig. 1). This suggests that geographical isolation exerts a significant effect on the genetic diversity of *Digitaria* species. Alternatively, this might have resulted from incorrect genotyping for several polyploid species that require high coverage to genotype correctly (Gould 1963; Koul and Gohil 1991; Kaur and Gupta 2016; Gerard *et al*. 2018). Published sequencing data for 8 *Digitaria* species were generated using target enrichment (Baker *et al.* 2021). The number of available SNPs is, therefore, limited and might not offer a comprehensive view of genomic diversity among *Digitaria* species. Phylogenetic analysis based on sequencing data for the entire genome of *Digitaria* species might provide more precise genetic classification of *Digitaria* species (Leaché and Oaks 2017).

We investigated the genetic structure of *Digitaria* species in Japan, among plants from the same species (intraspecific) and among different species (interspecific). As all *D. radicosa* plants were separated into 2 clusters by PCA, we speculate that the samples we collected are possibly composed of 2 genetically distinct subpopulations (Fig. 2b). The genetic difference based on 206 SNPs between these 2 clusters might not be associated with their geographical distribution, since plants collected from Shirahama were present into both clusters (Fig. 2b). In addition, plants collected from Kyoto and Kobe each formed a tight cluster, suggesting that these samples might be inbred or of clonal origin (Fig. 2b). Therefore, in Kyoto and Kobe, we might have sampled only 1 subpopulation, which does not preclude the existence of diverse subpopulations not yet sampled. The large genetic variation observed in Shirahama could be present also in other sampling locations.

*D. ciliaris* differentiates into divergent populations adapted to different environmental habitats (Kataoka *et al.* 1986; Tsuyuzaki 2005; Fukano *et al.* 2020). However, our results showed a loose clustering of *D. ciliaris* plant samples, suggesting the absence of clear population structure as a function of the environment (Fig. 2c). Therefore, in addition to parallel evolution of adaptive traits in each local population, frequent gene flow among different habitats might have also occurred in *D. ciliaris*. Gene flow fostered by the outcrossing capability of *D. ciliaris* may have maintained genetic variation of each locally adapted population under adaptive selection, as hypothesized for herbicide-resistant populations of several weed species, including *Digitaria insularis* (Kataoka and Kataoka 1991; Karn and Jasieniuk 2017; Gonçalves Netto *et al.* 2021; Brunharo and Tranel 2023).

In contrast to the unclear intraspecific genetic structure, we observed a clear species boundary between *D. radicosa* and *D. ciliaris*. As clusters comprising these 2 species were clearly distinguished with no intermediate samples, there is little possibility of interspecific hybridization (Fig. 2d). Difference in ploidy level may have worked as a reproductive barrier to keep these 2 species isolated (Hirayoshi and Yasue 1955).

We conclude that population genetic analysis based on our assembled reference genome hypothesized the existence of at least 2 distinct subpopulations in *D. radicosa*, which might not be associated with its geographical distribution. In addition, the different interspecific genetic structures of *Digitaria* species were highlighted. However, a more detailed analysis with a larger sample size will be required to unveil the population structure of *Digitaria* species across Japan.

Synteny analysis between *D. radicosa* and *D. exilis* identified a high degree of syntenic blocks across entire contigs and chromosomes, except for the possibility of 6 genome rearrangements (Fig. 3). Our evolutionary analysis showed that *D. radicosa* and *D. exilis* belong to different well-supported clades (Fig. 1). Therefore, these genome rearrangements might have occurred after the clades diverged. In addition, synteny analysis revealed that most rice genomic regions have syntenic blocks with some regions of the *D. radicosa* genome (Fig. 3). Since the genus *Digitaria* and the genus *Oryza* diverged almost 50 million years ago, this synteny may indicate that most core gene sets from Poaceae species are conserved in *D. radicosa* and rice (Pessoa-Filho *et al.* 2017). Comparative genomics including synteny analysis can help identify putative functions of genes (Bennetzen and Chen 2008; Wijerathna-Yapa *et al.* 2023). Therefore, the gene functions of Poaceae crops may be elucidated from these highly conserved synteny blocks between *D. radicosa* and Poaceae crops.

We identified NLR genes in the *D. radicosa* genome as representative resistance genes. *D. radicosa* has genes orthologous to rice *RGA4/5*, which recognize effectors secreted by *M. oryzae* and induce an immune response (Fig. 4) (Kanzaki *et al.* 2012; Cesari *et al.* 2013). Interestingly, the *RGA5* ortholog in *D. radicosa* carries the ID Exo70 in its C-terminus (Fig. 4). *RGA5* orthologs in *Oryza* species contain a wide variety of IDs and are thought to recognize corresponding effectors (Shimizu *et al.* 2022). In addition, Exo70 interacts with the *Pii-1/2* NLR pair to recognize the *M. oryzae* Avr-Pii effector to induce immune responses (Takagi *et al.* 2013; Fujisaki *et al.* 2017). Therefore, *RGA5* orthologs from *D. radicosa* may recognize effectors secreted by *M. oryzae* through their Exo70 domain. *D. radicosa* also has genes orthologous to a wide variety of functionally validated NLRs in Poaceae species (Supplementary Fig. 4). These results suggest that *D. radicosa* could be a useful genomic resource for improving plant immunity in Poaceae crops.

In summary, we constructed a chromosome-scale reference genome for the diploid species *D. radicosa*. This newly assembled diploid *Digitaria* genome allowed us to perform standard genomic analyses of *Digitaria* species. Our evolutionary analysis of 19 *Digitaria* species based on our new reference genome illustrated their extent of genetic relatedness. Population genetic analysis of *D. radicosa* and *D. ciliaris* revealed the genetic structure within species and the relationships between them. Larger-scale population genetic analysis will provide more valuable insights into the genetic relatedness of *Digitaria* species. Furthermore, the *D. radicosa* genome showed highly conserved syntenic blocks with the representative Poaceae crop and harbored putative resistance genes for *M. oryzae*. These findings indicate that our new diploid *Digitaria* genome could be a useful resource for genomic studies of *Digitaria* species and for Poaceae crop breeding.

## Supplementary Material

jkae242_Supplementary_Data

## Data Availability

PacBio HiFi sequencing reads and short reads generated for the reference genome have been deposited in the SRA of NCBI under BioProject PRJNA1097755. The assembled genome of *D. radicosa* was also deposited at NCBI under BioProject PRJNA1097755. MIG-seq sequencing data generated for population genetic analysis have been deposited in the SRA under BioProject PRJNA1098455. The sequences of the reference genome and gene annotation data were deposited in Zenodo (https://zenodo.org/doi/10.5281/zenodo.10952520). All scripts and commands used in this study were deposited in a GitHub repository (https://github.com/slt666666/D_radicosa_genome). Supplemental material available at G3 online.
