# Supplementary material for: A chromosome-scale genome assembly of Timorese crabgrass (Digitaria radicosa): a useful genomic resource for the Poaceae
Source: G3 (Bethesda). 2024 Oct 10;14(12):jkae242. doi: 10.1093/g3journal/jkae242 (PMC11631527; doi:10.1093/g3journal/jkae242)
Supplement: jkae242_Supplementary_Data [file jkae242_supplementary_data.zip › Supplemental_Figures_G3-2024-405351.pdf]

## Supplemental figures

# A chromosome-scale genome assembly of Timorese crabgrass (*Digitaria radicosa*): a useful genomic resource for the Poaceae

**Koki Minoji, Toshiyuki Sakai***

Laboratory of Crop Evolution, Graduate School of Agriculture, Kyoto University, Mozume, Muko, Kyoto 617-0001, Japan

* Corresponding author: Toshiyuki Sakai (sakai.toshiyuki.3w@kyoto-u.ac.jp)

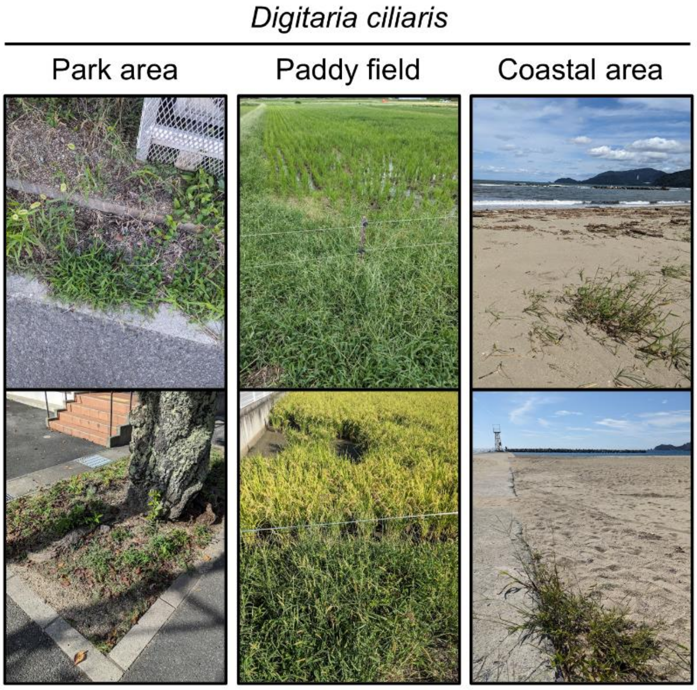


**Figure S1. Examples of *Digitaria ciliaris* plants growing in three habitats.** Representative examples of *D. ciliaris* growing in a park area (left), paddy field (middle), and coastal area (right).

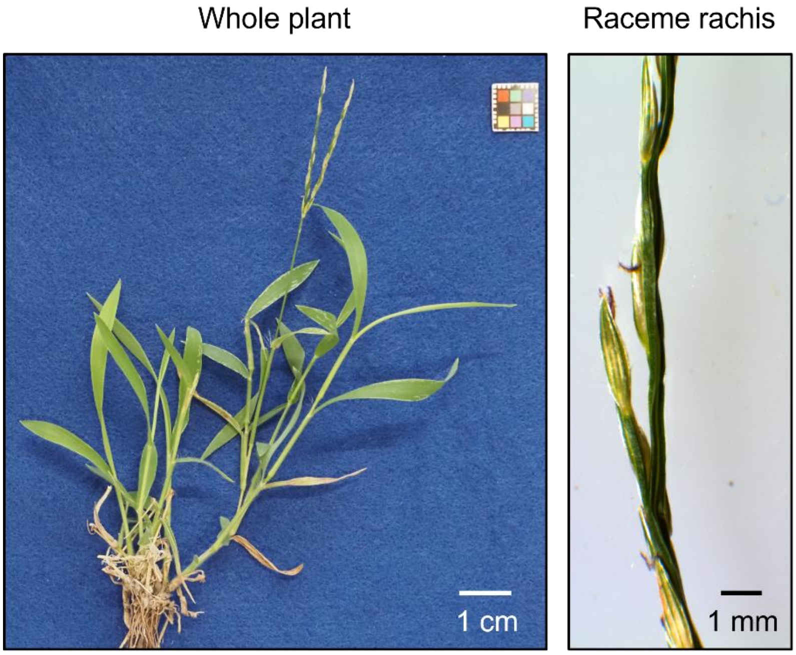

**Figure S2. Morphological characteristics of the *Digitaria radicosa* reference plant.** Representative example of *D. radicosa* and an image that zoomed raceme rachis.

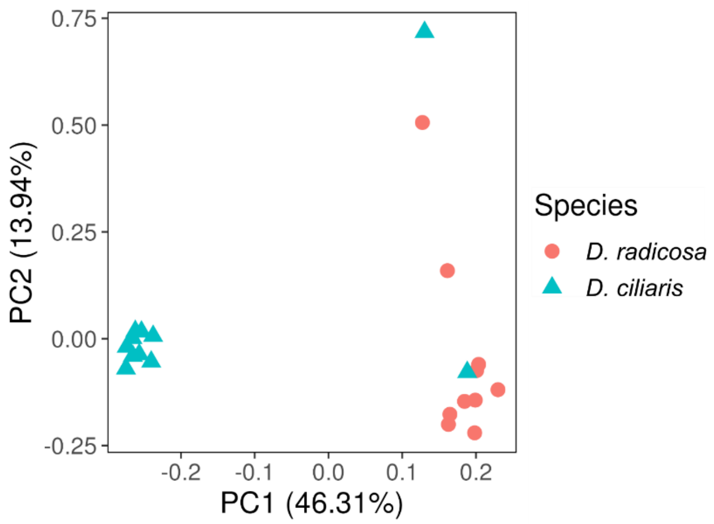


**Figure S3. Principal component analysis of all samples before removing putatively misidentified samples.** Two *D. ciliaris* samples clustered with the *D. radicosa* samples and were suspected to have been misidentified as *D. ciliaris*.

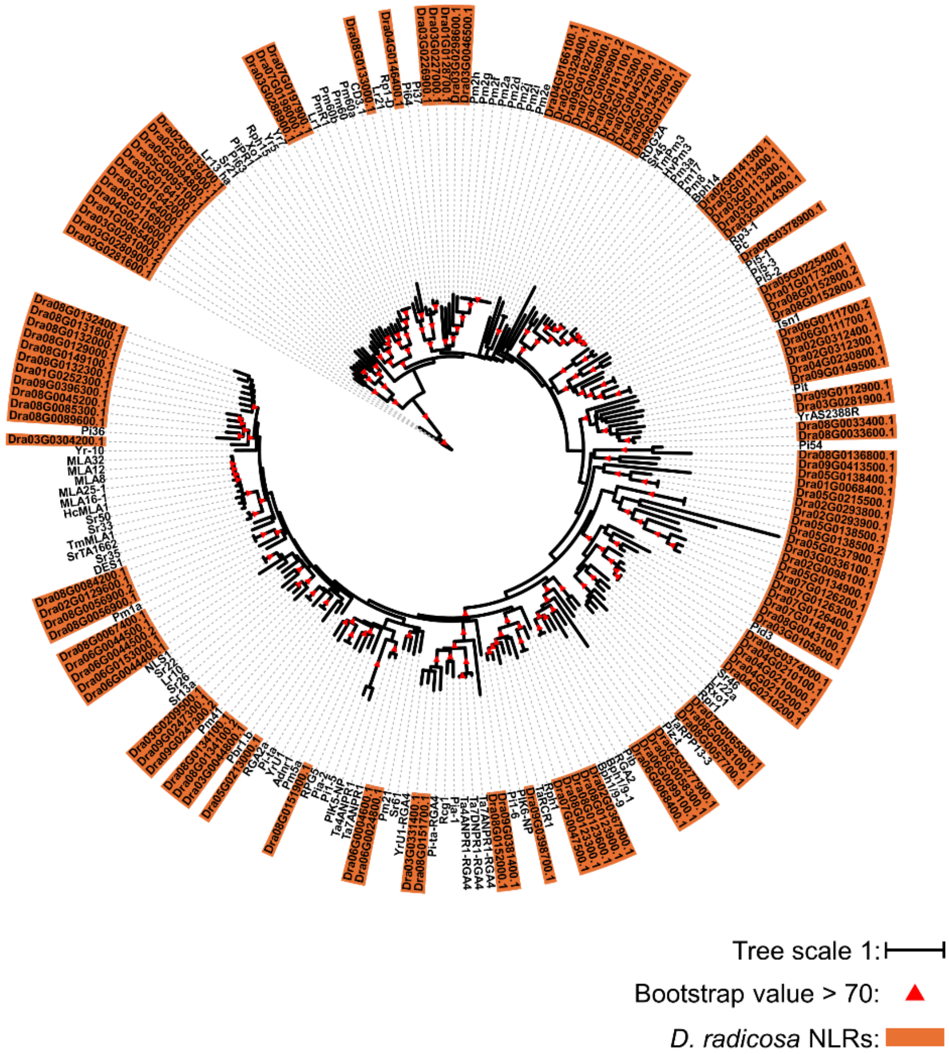


**Figure S4. Phylogenetic tree of identified NLR proteins from *Digitaria radicosa* and functionally validated NLRs from Poaceae species.** Phylogeny of 126 NLR proteins from *D. radicosa* and 100 functionally validated NLR proteins from Poaceae species. The maximum-likelihood phylogenetic tree was generated in RAxML version 8.2.12 with the JTT model using the amino acid sequences of NB-ARC domains. *D. radicosa* NLRs are highlighted in orange. The red arrowheads indicate bootstrap support > 0.7 based on 100 iterations. The scale bar corresponds to the mean number of amino acid substitutions per site on the respective branch.

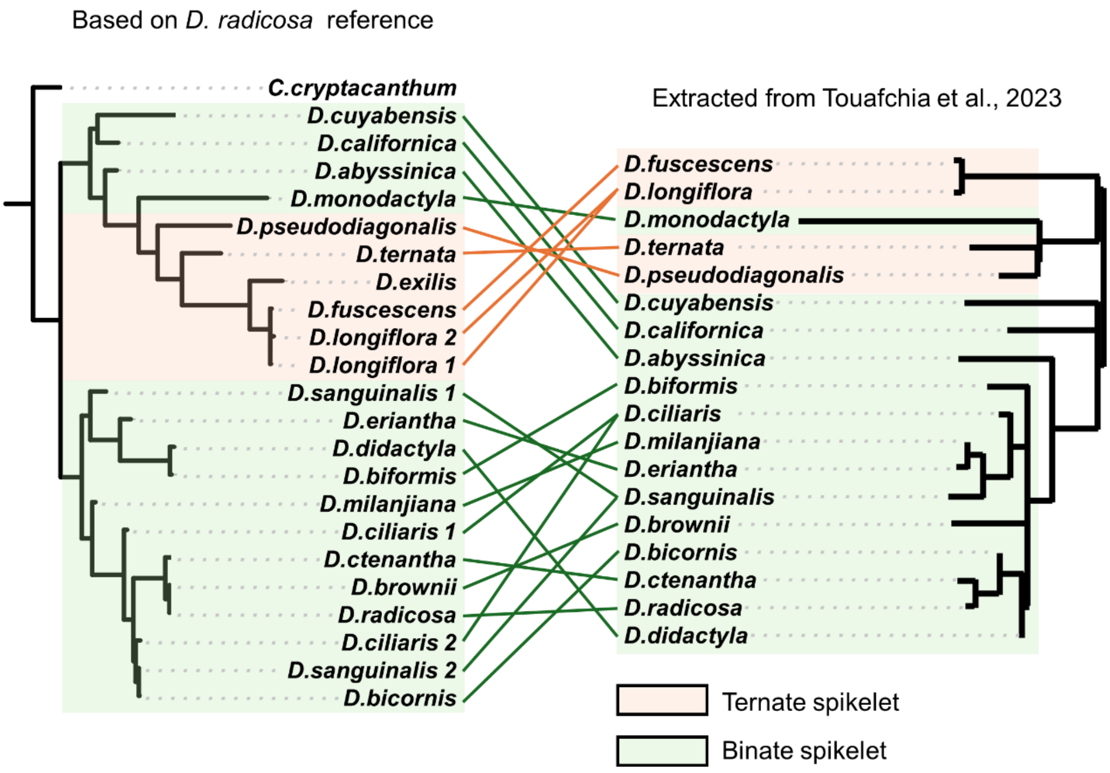


**Figure S5. Comparison of our phylogenetic tree of Digitaria species with previously reported one.** Phylogenetic tree of 19 *Digitaria* species and one *Chlorocalymma* species as an outgroup. The left maximum-likelihood phylogenetic tree was generated in RAxML version 8.2.12 with the JTT model using 2,052 SNPs. The right tree was extracted from Figure3 in Touafchia *et al*., 2023. The number of spikelets at each node on the rachis is indicated by different background colors.
